# Improving Recruitment and Retention by Updating and Developing a More Comprehensive Prospectus to Enhance NEL CAP Trainee Confidence: A Quality Improvement Project

**DOI:** 10.1192/bjo.2026.11493

**Published:** 2026-06-30

**Authors:** Anujavahinie Suntharamoorthy, Darshana Arakkal

**Affiliations:** 1 East London NHS Foundation Trust (ELFT), London, United Kingdom; 2 Great Ormond Street Hospital (GOSH), London, United Kingdom

## Abstract

**Aims:**

The training scheme prospectus is usually the initial reference for prospective Higher Trainees (HT) in ranking training programmes. An outdated prospectus fails to showcase the strength of individual training schemes, resulting in failure to attract prospective trainees. The North Central and East London (NEL) Prospectus for Child and Adolescent Psychiatry (CAP) on the Health Education England (HEE) website had not been updated since2021 and recruitment into the scheme had declined resulting in up to 40% vacancies for the February 2025 recruitment cycle.

The aim of this QIP is to revise and update the NEL CAP training scheme prospectus to improve recruitment and retention of HTs by enhancing trainee confidence.

**Methods:**

A PDSA cycle using surveys and focus groups including trainees and trainers at each placement across 4 Trusts, was used to gather feedback to identify gaps in the Prospectus. The key areas identified for revision included providing detailed placement descriptions and job-specific teaching, research, psychotherapy, special interest and leadership opportunities.

**Results:**

An initial survey of the trainees in the last 3 years indicated that more than half of the trainees (53%) never referred to the outdated Prospectus to guide their scheme selection to inform placement ranking, or to explore training opportunities. Of the 47% who referred to the prospectus, only 31% felt that their scheme selection was guided by the prospectus. Only 33% of the trainees used the outdated prospectus to rank their placements and to explore job-specific special interest opportunities.

Following the initial survey, the updated prospectus was distributed to all the trainees in February 2026. Formal qualitative feedback from trainees showed that the updated job descriptions of posts ‘reads well’, ‘pitched at a good level of detail’, shows ‘what to expect’,‘eases anxieties’,helped them ‘understand their roles’,and provides job-specific opportunities. Consultant feedback suggested that the updated prospectus was necessary in reflecting the strength of the training scheme and the broad range of opportunities available to the trainees.

The revised prospectus will be shared with HEE and the second cycle of PDSA will be completed in the next recruitment cycle in August 2026.

**Conclusion:**

The revised prospectus has improved trainee confidence in the Scheme and in guiding their training progression. Further cycles will evaluate its impact on trainee recruitment and retention to the scheme.

